# Prejunctional and postjunctional actions of heptanol and 18β-glycyrretinic acid in the rodent vas deferens

**DOI:** 10.1016/j.autneu.2009.03.006

**Published:** 2009-06-15

**Authors:** Faisal Rahman, Rohit Manchanda, Keith L. Brain

**Affiliations:** a Department of Pharmacology, University of Oxford, Mansfield Rd., Oxford, OX3 0RP, UK; b School of Biosciences and Bioengineering, Indian Institute of Technology Bombay, Powai, Mumbai 400 076, India

**Keywords:** Heptanol, 18β-glycyrretinic acid, Sympathetic neurotransmission, Gap junctions, Smooth muscle

## Abstract

Heptanol and 18β-glycyrrhetinic acid (18βGA) block gap junctions, but have other actions on transmitter release that have not been characterised. This study investigates the prejunctional and postjunctional effects of these compounds in guinea pig and mouse vas deferens using intracellular electrophysiological recording and confocal Ca^2+^ imaging of sympathetic nerve terminals. In mice, heptanol (2 mM) reversibly decreased the amplitude of purinergic excitatory junction potentials (EJPs; 52 ± 5%, *P* < 0.05) while having little effect on spontaneous excitatory junction potentials (sEJPs). Heptanol (2 mM) reversibly abolished the nerve terminal Ca^2+^ transient in 52% of terminals. 18βGA (10 μM) decreased the mean EJP amplitude, and increased input resistance in both mouse (137 ± 17%, *P* < 0.05) and guinea pig (354 ± 50%, *P* < 0.001) vas deferens indicating gap junction blockade. Further, 18βGA increased the sEJP frequency significantly in guinea pigs (by 71 ± 25%, *P* < 0.05) and in 5 out of 6 tissues in mice (19 ± 3%, *P* < 0.05). Moreover, 18βGA depolarised cells from both mice (11 ± 1%, *P* < 0.01) and guinea pigs (8 ± 1%, *P* < 0.005). Therefore, we conclude that heptanol (2 mM) decreases neurotransmitter release (given the decrease in EJP amplitude) by abolishing the nerve terminal action potential in a proportion of nerve terminals. 18βGA (10 μM) effectively blocks the gap junctions, but the increase in sEJP frequency suggests an additional prejunctional effect, which might involve the induction of spontaneous nerve terminal action potentials.

## 1 Introduction

Gap junctions are complexes that form hydrophilic channels between neighbouring cells. By allowing the passage of ions and small molecules between cells, gap junctions provide a mechanism of intercellular communication (for review, see de Wit and colleagues, 2006). Gap junctions are present in many tissues including smooth muscle, endothelial cells, glia (both central and peripheral), cardiac muscle and some neurons (for review, see Sohl et al., 2005). Obtaining specific blocker of gap junctions is therefore of great interest in order to study the communication between cells and potentially for clinical therapies (such as for overactive bladder syndrome)(Christ et al., 2003). Agents that block gap junctions include: long-chain alcohols (e.g. 1-heptanol, 1-octanol), glycyrrhetinic acid (18α and 18β isoforms), carbenoxolone, connexin mimetic peptides (e.g. gap 27), connexin antibodies (Mather et al., 2005), retinaldehyde (Pulukuri and Sitaramayya, 2004), organochlorine pesticides, and general anaesthetics (e.g. halothane). However, target specificity is important if such drugs are to be used in tissues containing more than one cell type, such as smooth muscle and its innervating nerve terminals.

Nerve terminals in the vas deferens release the neurotransmitters ATP and noradrenaline upon stimulation. ATP activates purinergic P2X receptors (ligand-gated cation channels) to produce an excitatory junction potential (EJP) (Sneddon and Machaly, 1992). In addition, spontaneous excitatory junction potentials (sEJPs) are produced by the spontaneous release of packets of ATP from varicosities in the absence of stimulation. In the guinea pig vas deferens, not every smooth muscle cell is directly innervated (Merrillees, 1968) and not every nerve action potential triggers release of neurotransmitters by every varicosity (Cunnane and Stjärne, 1984), so gap junctions are important in the spread of excitation from directly activated cells to cause EJPs and sEJPs in cells not directly innervated (Venkateswarlu et al., 1999).

Several studies argue that heptanol and 18βGA selectively block gap junctions (Christ, 1995; Manchanda and Venkateswarlu, 1997) while others have questioned the mechanism of action on smooth muscle cells (Chaytor et al., 1997; Tare et al., 2002; Yamamoto et al., 1998). Much less is known of the actions of these drugs on nerve terminals. The aim of the present study was to investigate the possibility of prejunctional as well as postjunctional effects of these agents in mouse and guinea pig vas deferens.

## 2 Materials and methods

### 2.1 Tissue preparation

All experimental procedures were in accordance with the UK Animals (Scientific Procedures) Act 1986. Male guinea pigs and mice (Balb/C) were killed by concussion and cervical fracture. Using a midline abdominal incision both vasa deferentia were dissected out. The connective tissue overlying the vasa deferentia was carefully removed under a dissecting microscope. Each vas deferens was immobilised by pinning it to the Sylgard-covered base of a Perspex organ bath. The tissue was allowed to stabilise for 30 min following pinning. Preparations were immersed in Krebs solution and oxygenated by continuous bubbling of the solution with 95% O_2_, 5% CO_2_ to maintain a pH of 7.4. The solution was warmed to 33 ± 1 °C. The Krebs solution contained (mM): NaCl 118.4, NaHCO_3_ 25.0, NaH_2_PO_4_ 1.13, CaCl_2_ 1.8, KCl 4.7, MgCl_2_ 1.3 and glucose 11.1.

### 2.2 Intracellular recordings

Standard intracellular recording procedures were used to measure membrane potential (*E*_m_) changes in vas deferens smooth muscle cells. Briefly, this involved using glass microelectrodes filled with 5 M potassium acetate and with resistances between 40–100 MΩ. The microelectrodes were connected to the high input impedance headstage of an Axoclamp 2A (Axon Instruments, Sunnyvale, CA, USA). *E*_m_ measurements were digitised with a PowerLab system and stored on a Macintosh computer (using Chart 5.0, ADI Instruments, Chalgrove, UK) for subsequent analysis. The criteria for a successful impalement of a smooth muscle cell were: a rapid change in potential upon impalement and withdrawal, *E*_m_ more negative than − 60 mV, and the presence of spontaneous excitatory junction potentials (sEJPs) during recordings. The resting *E*_m_ was measured as the difference between the recorded voltage inside the cell after impalement and outside the cell after withdrawal of the electrode.

Intrinsic nerves were stimulated by a pair of parallel electrodes placed around the prostatic third of the preparation. Mouse vas deferens was stimulated by sequences of 5 rectangular voltage pulses (5 Hz, 0.1 ms duration, 10–20 V). Due to the difficulty of maintaining intracellular insertions, guinea pig vas deferens received trains of only 2 pulses at a lower frequency of 1 Hz (0.6 ms, 10–20 V). At least 4 trains of stimuli were given to each preparation with 20 s intervals between each train.

### 2.3 Input impedance

Input resistance (*R*_in_) was determined from Ohm's Law by measuring the steady-state change in membrane potential following injection of a 0.5 nA current. The *R*_in_ depends on the membrane resistance (*R*_m_) and junctional resistance (*R*_j_): 1/*R*_in_ = 1/*R*_m_ + 1/*R*_j_. In well-coupled tissues, where *R*_m_ >> *R*_j_, the *E*_m_ change upon current injection should effectively reflect changes in junctional resistance and should provide an estimate of the extent of cell coupling (Purves, 1976). Blocking gap junctions will increase *R*_j_ and therefore *R*_in_. Although cable potentials would provide a more precise measurement of this parameter, intracellular recording has the advantage of allowing a simultaneous measure of transmitter release.

### 2.4 Ca^2+^ imaging

Nerve terminals in the mouse vas deferens were orthogradely filled with the Ca^2+^ indicator Oregon Green 488 BAPTA-1 AM 10 kDa dextran (OGB-1; Invitrogen, Paisley, UK) as previously described (Brain and Bennett, 1997). To summarise: after removing each vas deferens (as above), the cut prostatic end was exposed to a 0.25 mg.µl^− 1^ solution of OGB-1 for 8 h, rinsed in Krebs' solution for 2 h, then transferred to a Leica TCS NT inverted confocal microscope (Leica Microsystems, Milton Keynes, Buckinghamshire, UK). Excitation illumination (with an Ar ion laser) was at 488 nm; the emission was sampled with a long pass 510 nm filter. A 40× water immersion objective was used to obtain images of a field 158 μm^2^ at a sampling rate just slower than 2 Hz. Field stimuli were applied with electrodes embracing the tissue (as described above) and were synchronised by a TTL signal from the microscope software to occur at the start of every 4th confocal scan (giving a stimulus rate of around 0.5 Hz). The field size and sampling rate were chosen to increase the number of terminals recorded at the expense of some spatial and temporal resolution. Images were acquired in sets of 42 frames (10 stimuli), with 3 such sets acquired for each experimental condition (30 stimuli). Drugs were applied by changing the solution that continuously superfused the organ bath on the microscope stage (bath exchange time was 1 min). Image analysis was with Image SXM (http://www.liv.ac.uk/~sdb/ImageSXM/) and custom-written macros. Fluorescent signals were averaged over regions of interest (ROI) manually selected to include a single varicose terminal; lateral movement was corrected by locally tracking this ROI with an automated algorithm, as previously described (Brain and Bennett, 1997).

No reliable protocol to load the Ca^2+^ indicator into terminals in the guinea pig vas deferens has, as yet, been developed.

### 2.5 Drugs

Heptanol was used at 2 mM (final concentration) similar to previous studies of gap junctions (Manchanda and Venkateswarlu, 1997; Manchanda and Venkateswarlu, 1999). Although some studies use 30–40 μM 18βGA (Matchkov et al., 2004; Yamamoto et al., 1998), 10 μM seems to be sufficient to inhibit gap junctions (Takeda et al., 2005). Therefore, in order to minimise non-specific actions at high concentrations, 18βGA was used at a 10 μM final concentration after dilution from a 10 mM stock solution in dimethyl sulfoxide (DMSO). The final concentration of DMSO was 0.1%. The tissue was exposed to the drug for 30 min to allow the drug to take effect before recordings were made. All drugs were obtained from Sigma-Aldrich (Dorset, UK).

### 2.6 Statistics

The assumptions of a normal distribution and homogeneity of variances were checked with the Kolmogorov–Smirnov Test and Levene Test, respectively, prior to parametric tests. Statistical significance was tested using a paired Student's *t*-test, pairing pre- and post-drug responses in the same preparation. Probabilities less than 0.05 (*P* < 0.05) were taken as statistically significant. *n*_c_ refers to the number of pairs of cells; *n*_p_ refers to the number of preparations. Data here are reported as mean ± S.E.M. (standard error of the mean) using *n*_p_ for calculations. In addition, the variability of the EJP amplitude can also be used as a test for electrical coupling (Young et al., 2007). To assess this parameter, the *F*-test was used to check if the standard deviation of the EJP amplitudes in control and in the drug were equal after the EJP amplitudes were normalised. All statistical tests were performed with Prism (GraphPad, Software, San Diego, CA, USA).

## 3 Results

### 3.1 *R*_in_ and membrane potential

**Fig. 1:**
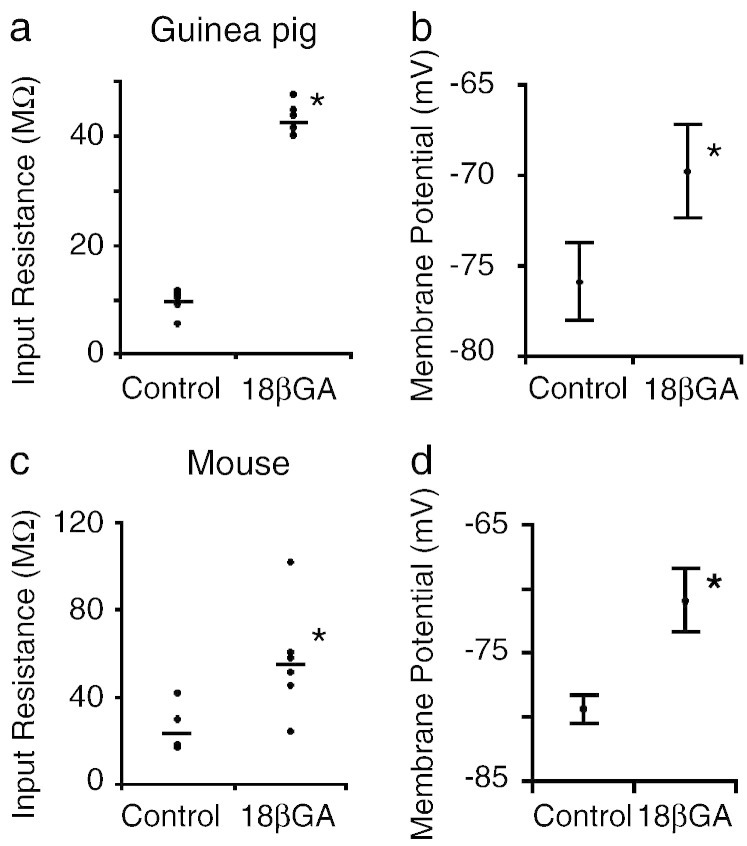
The effect of 18βGA on cell input resistance and membrane potential. 18βGA (10 μM) increased both the input resistance (a, c) and resting membrane potential (b, d) in both guinea pig (*n* = 6; a, b) and mouse (*n* = *6*; c, d) vas deferens. In the input resistance graphs (a, c), the bars represent the mean. ⁎ denotes *P* < 0.05 compared with the control.

The effect of 18βGA (10 μM) on *R*_in_ was tested on both mouse and guinea pig vas deferens. A partially reversible increase in *R*_in_ was observed in guinea pig (by 354 ± 50%; control: 9.6 ± 1.4 MΩ, 18βGA: 43.6 ± 1.5 MΩ; *n*_c_ = 26, *n*_p_ = 5, *P* < 0.001; Fig. 1a) and mouse (by 137 ± 17%, control: 24.1 ± 4.5 MΩ, 18βGA: 57.0 ± 11.3 MΩ; *n*_c_ = 36, *n*_p_ = 6, *P* < 0.05; Fig. 1c) vas deferens. 18βGA also significantly depolarised cells in guinea pig (by 8 ± 1%, control: − 75.8 ± 2.1 mV, 18βGA: − 69.8 ± 2.6 mV; *n*_c_ = 36, *n*_p_ = 6, *P* < 0.005; Fig. 1b) and mouse (by 11 ± 1%, control: − 79.4 ± 1.1 mV, 18βGA: − 70.9 ± 2.5 mV; *n*_c_ = 35, *n*_p_ = 6, *P* < 0.01; Fig. 1d) vas deferens.

Unlike 18βGA (10 μM), heptanol (2 mM) in mice did not affect resting *E*_m_. The mean *E*_m_ in control (− 79 mV) and with heptanol (− 78 mV) was not significantly different.

### 3.2 Spontaneous EJPs (sEJPs)

Changes in cell-to-cell coupling affecting the spread of current between cells should produce changes in sEJP shape and amplitude, as explained in the discussion. In the mouse, heptanol (2 mM) did not significantly change sEJP amplitude, frequency or time to fall from 90% to 50% of peak (*F*_90–50_; median amplitude 1.93 ± 0.32 to 1.96 ± 0.35 mV; frequency 0.188 ± 0.034 to 0.189 ± 0.024 Hz; fall time 21.0 ± 0.6 to 20.7 ± 0.7 ms; *n*_p_ = 6).

**Fig. 2:**
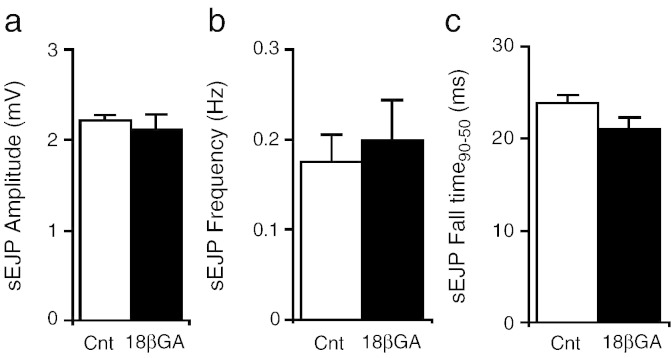
The effects of 18βGA on sEJPs in mouse vas deferens. 18βGA (10 μM) had no effect on sEJP (a) amplitude, (b) frequency or (c) *F*_90–50_ in the mouse vas deferens.

**Fig. 3:**
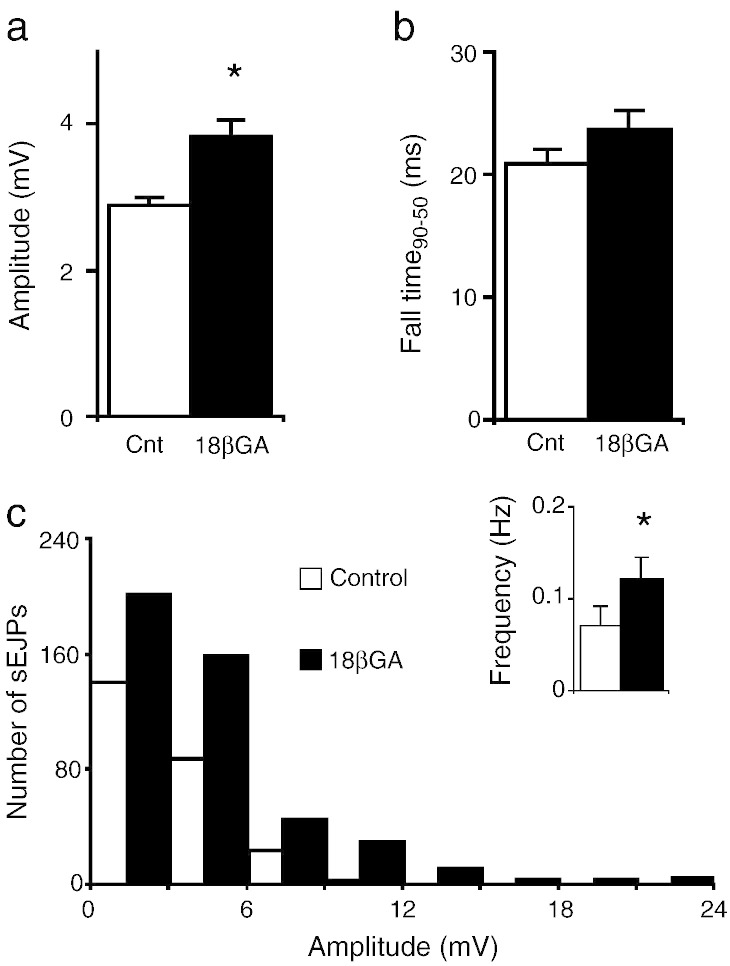
The effects of 18βGA on sEJPs in guinea pig vas deferens. The effects of 18βGA (10 μM) on sEJP (a) amplitude and (b) *F*_90–50_. (c) shows the amplitude histogram of sEJPs illustrating an increase in sEJP frequency (pooled across all frequencies in the inset) and the occurrence of high amplitude sEJPs. The inset shows the change in frequency pooled across all sEJP amplitudes. ⁎ denotes *P* < 0.05 compared with control.

Similarly, 18βGA (10 μM) did not change sEJP characteristics significantly in the mouse (Fig. 2). However, in 5 of 6 preparations there was a significant increase in sEJP frequency (19 ± 3%, *n*_c_ = 30, *n*_p_ = 5, *P* < 0.05). In guinea pig vas deferens 18βGA administration also increased sEJP amplitude (by 34 ± 4%, *n*_c_ = 36, *n*_p_ = 6, *P* < 0.05; Fig. 3a). Associated with this change was an increase in high amplitude sEJPs (Fig. 3c) and a statistically significant increase in sEJP frequency (by 71 ± 25%; control: 0.07 ± 0.02 Hz, 18βGA: 0.12 ± 0.02 Hz; *n*_c_ = 36, *n*_p_ = 6, *P* < 0.05; Fig. 3i). On the other hand, the time course of the sEJP, assessed as the *F*_90–50_, was not significantly affected (Fig. 3b).

### 3.3 EJPs

**Fig. 4:**
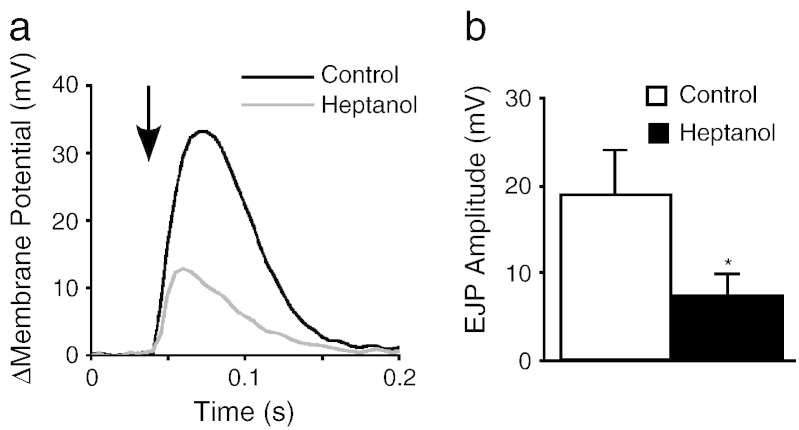
The effect of heptanol on EJPs in guinea pig vas deferens. There was a decrease in EJP amplitude on exposure to heptanol (2 mM) in mice. (a) shows representative EJPs before and after heptanol exposure. (b) shows that the mean EJP amplitude decreased in the presence of heptanol (*n* = 6). A single field stimulus was applied at the arrow. ⁎ denotes *P* < 0.05 compared with control.

Heptanol (2 mM) reversibly decreased average EJP amplitude in mouse vas deferens (by 60 ± 5%; control: 19.0 ± 5.1 mV, heptanol: 7.5 ± 2.4 mV, *n*_c_ = 36, *n*_p_ = 6, *P* < 0.05; Fig. 4). Paired-pulse facilitation was calculated as the ratio of the amplitude of the second stimulus to the first stimulus (no example is shown). No significant change in the facilitation was observed after heptanol. Further, the latency from stimulus to EJP peak was reversibly increased by heptanol (by 27 ± 5%, *n*_c_ = 36, *n*_p_ = 6, *P* < 0.05). Although a consistent effect on the variability of EJP amplitude was not found, 4 of the 6 tissues did show a significant (*P* < 0.05) increase in variability of the first stimulus EJP amplitude using the *F*-test.

**Fig. 5:**
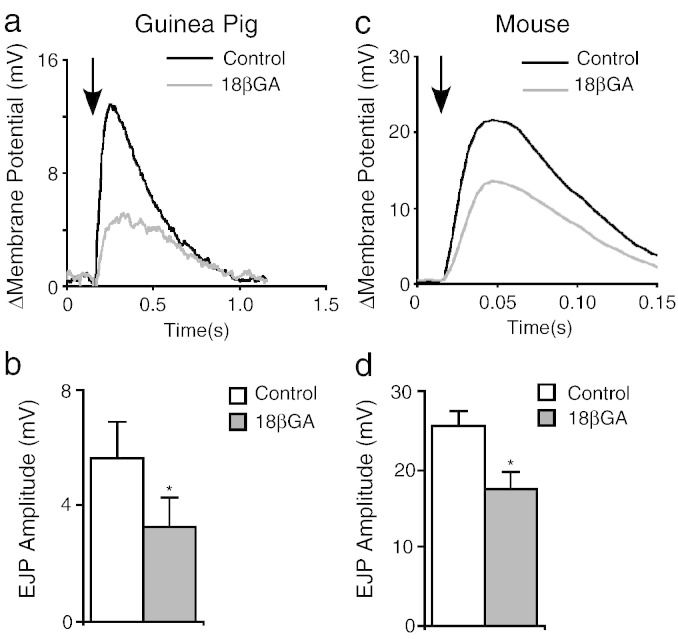
Decreased EJP amplitude in 18βGA from guinea pig and mouse vas deferens. Sample traces of EJP amplitude before and after 18βGA in (a) guinea pig and (b) mouse vas deferens. Mean EJP amplitude decreased in (c) guinea pig (*n* = 6) and (d) mouse (*n* = 6) vas deferens. A single field stimulus was applied at the arrow. ⁎ denotes *P* < 0.005 compared with control.

Similarly, 18βGA (10 μM) decreased EJP amplitude in guinea pig (by 44 ± 3%, control: 5.7 ± 1.2 mV, 18βGA: 3.2 ± 1.0 mV; *n*_c_ = 36, *n*_p_ = 6, *P* < 0.005; Fig. 5a, b) and mouse (by 32 ± 3%, control: 25.6 ± 1.9 mV, 18βGA: 17.3 ± 2.5 mV; *n*_c_ = 36, *n*_p_ = 6, *P* < 0.005; Fig. 5c, d) vas deferens. In the guinea pig the EJP was almost completely abolished upon stimulation in several cells. Upon washout the effect of 18βGA was not reversed and EJP amplitude remained significantly smaller than in control in both mouse (by 25 ± 3%, *P* < 0.05) and guinea pig (by 34 ± 7%, *P* < 0.05) vas deferens. Analysis of facilitation and the stimulus peak latency showed no significant change in either species. A significant, irreversible decrease in the time constant of decay of the EJP was found in guinea pig vas deferens (by 35 ± 2% *P* < 0.005). In mouse (4/6 tissues), and guinea pig (5/6 tissues) vas deferens, there was a significant increase in the variability of the EJP amplitude.

### 3.4 Nerve terminal Ca^2+^ imaging

**Fig. 6:**
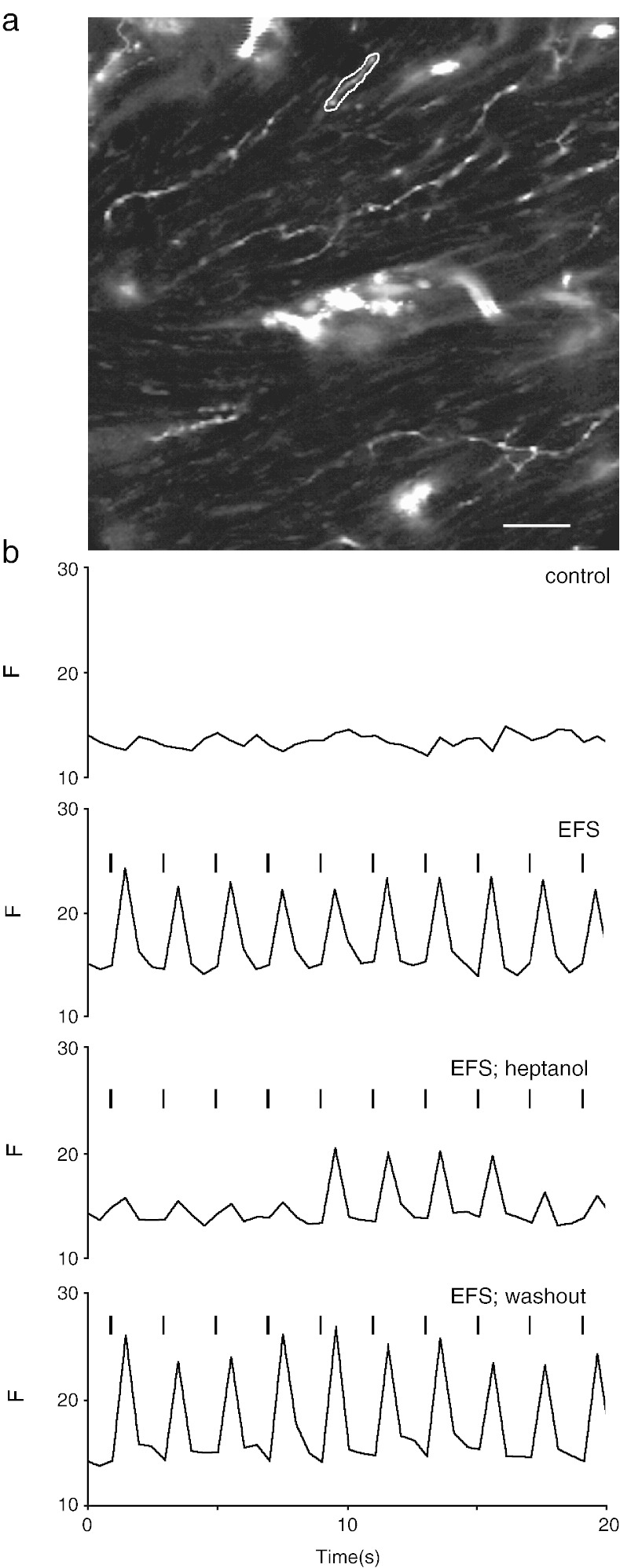
The effect of heptanol on nerve terminal Ca^2+^. a, shows a field of OGB-1 loaded nerve terminals in the mouse vas deferens. A sample ROI has been drawn in white around a terminal in the upper part of the figure. The scale bar is 20 μm. b, shows sample recordings of the fluorescent signal (*F*) from the marked ROI under control conditions, then during electrical field stimulation (EFS; with each stimulus marked with a vertical bar), EFS in the presence of heptanol (2 mM), and then subsequent EFS once heptanol had been washed from the organ bath. In this terminal the Ca^2+^ signal is of intermittent or highly-variable amplitude in the presence of heptanol.

Potential prejunctional effects of heptanol (2 mM) and 18βGA (10 μM) were further investigated by imaging the Ca^2+^ concentration in the nerve terminals of the mouse vas deferens. The fluorescent signal from each nerve terminal (*F*; see Fig. 6A for an example of the region sampled) increased on each field (nerve) stimulus (Fig. 6B; ‘EFS’). The value of *F* immediately after the stimulus was compared to the trough immediately before it (*F*_o_); their difference (Δ*F*) was normalised (Δ*F*/*F*_o_) to give a relative measure of the change in Ca^2+^ concentration in a nerve terminal ([Ca^2+^]_t_). Heptanol (2 mM) either abolished the Ca^2+^ transient (13 of 25 terminals from 3 of 4 vasa deferentia; 52%), or caused intermittent evoked Ca^2+^ transients (12 of 25 terminals from 4 of 4 vasa deferentia; 48%). The Ca^2+^ transients did not return (nor become less variable) by increasing the amplitude of the field stimulus to 50 V, suggesting that the stimulus threshold for initiating nerve terminal action potentials had not changed, at least over the range tested. In those terminals still intermittently responding, the mean probability of response per stimulus was 0.42 ± 0.06 (*n* = 12 terminals) and the amplitude of such responses was 78 ± 5% of the control amplitude (paired two-tailed *t*-test; *P* < 0.05). After washing out the bath for 10 min the Ca^2+^ transients partially returned (except in one terminal) with a mean probability of response per stimulus of 0.72 ± 0.06 and such responses were of the same amplitude as the control (103 ± 5%; *n* = 24 terminals; paired *t*-test, *P* = 0.46).

Using the same exposure protocol, 18βGA (10 μM) had no significant effect on the [Ca^2+^]_t_ in response to field stimuli. The Ca^2+^ transients did not become intermittent and the amplitude remained unchanged at 106 ± 3% of the control amplitude (*n* = 21 terminals from 4 vas deferens; *P* = 0.11 with a Wilcoxon signed rank test). However, in the presence of 18βGA 4 of these terminals (all from 1 preparation; see Supplementary Movie) showed spontaneous whole-terminal Ca^2+^ transients of similar amplitude to those observed following field stimulation. Such spontaneous transients were never observed during the control recording (for any experimental protocol).

## 4 Discussion

The present study shows that both heptanol (2 mM) and 18βGA (10 μM) have significant prejunctional effects on sympathetic terminals.

### 4.1 Heptanol

Early support for heptanol's uncoupling effects came from its ability to block the transfer of fluorescent dye between smooth muscle cells (Christ, 1995). In agreement with the work of Manchanda and Venkateswarlu (1997), this study shows that heptanol (2 mM) reduces EJP amplitudes (Fig. 4). Since not all cells receive innervation, at least in the guinea pig (Merrillees, 1968), and only some varicosities release neurotransmitters on each nerve action potential (Cunnane and Stjärne, 1984), most EJPs are thought to be initiated in cells neighbouring the monitored cell. Due to electrical coupling depolarising current can spread to generate an EJP in the cell being monitored. Therefore, by blocking gap junctions, heptanol could prevent the spread of depolarisation and thus reduce the EJP amplitude. However, inhibition of neurotransmitter release could also reduce EJP amplitude (Manchanda and Venkateswarlu, 1997). The present work suggests that the EJP amplitude is reduced because of intermittent, or complete, abolition of the Ca^2+^ transient in the nerve terminals, probably due to failure of action potential propagation in the axons or terminals. This is also supported by the finding of increased latency of the EJP peak, which would arise in the presence of slowed nerve terminal action potential velocity.

sEJP properties are good, if indirect, indicators for changes in gap junctional coupling (Ghildyal et al., 2006). Gap junctions provide low resistance pathways for current flow between cells (Purves, 1976). Good electrical coupling is well established in the guinea pig vas deferens, while in the mouse there is a small population of poorly-coupled cells, with the rest of the cells being well electrically coupled (Blakeley et al., 1989; Young et al., 2007). The mouse vas deferens smooth muscle cells impaled in the present study had relatively negative membrane potentials and EJPs of regular amplitude, consistent with them being from the relatively well-coupled population (Blakeley et al., 1989). Therefore, it is expected that by decreasing the shunting of current (by blocking gap junctions) the sEJP fall time would increase. Additionally, as most low amplitude sEJPs are generated in cells other than the one being recorded from, the frequency should decrease as sEJPs generated in other cells become part of the recording noise (Palani et al., 2006). When gap junctions are blocked, the amplitude of sEJPs generated in the recording cell should increase, current being unable to spread to neighbouring cells. However, the amplitude of sEJPs generated in neighbouring cells should decrease (in the limiting case, disappearing), as the high intervening electrical resistance damps their amplitude at the recording electrode. However, the amplitude, frequency and F_90−50_ did not change significantly after heptanol (2 mM) administration, which is inconsistent with an uncoupling effect. Further, since sEJPs are produced by ATP activation of purinergic receptors, the absence of an effect on sEJP amplitudes suggests that heptanol does not act on P2X receptors.

In agreement with Palani and Manchanda (2006), heptanol (2 mM) did not affect resting *E*_m_ of vas deferens smooth muscle in the present study. However, in vascular smooth muscle both hyperpolarisation (Lagaud et al., 2002) and depolarisation (Matchkov et al., 2004) have been reported. Effects on resting *E*_m_ may be preparation, concentration (Matchkov et al., 2004), or species dependent. However, all the studies report a reduction in EJP amplitude or smooth muscle tension suggesting that any resting smooth muscle *E*_m_ effects of heptanol do not govern the effects on the EJP and smooth muscle contraction.

Reports of *R*_in_ using heptanol (2 mM) are variable and not significant in the guinea pig vas deferens (Manchanda and Venkateswarlu, 1999) suggesting that cell uncoupling may not be occurring effectively; the effect on *R*_in_ has not been reassessed in the present study.

One of us has previously reported that in the guinea pig vas deferens, heptanol reduces the amplitude of the EJPs without affecting the amplitude of the nerve terminal impulse (an extracellular measure of the nerve terminal action potential) and excitatory junction currents (an extracellular measure of neurotransmitter release) from which it was inferred that heptanol did not affect nerve impulse conduction (Manchanda and Venkateswarlu, 1997), although subsequent wavelet analysis of heptanol's effect argued that heptanol did inhibit neurotransmitter release. Whether the effects of heptanol on the nerve terminal Ca^2+^ transients in a proportion of terminals from the mouse vas deferens implies that the mouse and guinea pig differ in the nature of their response to heptanol, or whether the nerve terminal impulse (which might represent a compound action potential in small bundles of axons known to run across the surface of this tissue) might not have sufficient sensitivity to detect heptanol's action in only a proportion of terminals, has not been determined. Furthermore, the amplitude and shape of intermittent excitatory junction currents are not expected to change with intermittent action potential abolition, although the frequency of such excitatory junction currents might drop.

### 4.2 18β-glycyrrhetinic acid

18βGA (10 μM) is usually considered a more specific uncoupling agent than heptanol (2 mM) (Griffith, 2004; Guan et al., 2007; Tare et al., 2002). Gap junctions provide low-resistance shunts for injected current, thus uncoupling should increase *R*_in_. Fig. 1 shows that 18βGA doubled *R*_in_ in mice and increased *R*_in_ by over three-fold in guinea pigs indicating gap junction blockade. Using the values of *R*_in_, *R*_m_ and time constant (*τ*) from Bywater and Taylor (1980), Blakeley et al. (1989) and this study, calculations show that such large changes in *R*_in_ in both tissues can be explained only by an increase in *R*_j_ (see Appendix A).

Interestingly, the much greater increase in *R*_in_ in guinea pig vas deferens suggests that cell coupling is more important in the guinea pig than in the mouse, consistent with previous reports (Brock and Cunnane, 1992).

Like heptanol (2 mM), 18βGA (10 μM) decreased EJP amplitude in both species. As noted above, this does not necessarily indicate a gap junctional effect. However, 18βGA increased the variability of the EJP amplitudes in 4 out of 6 tissues in mice and 5 out of 6 tissues in guinea pigs. Gap junction uncoupling is expected to increase variability due to the increase in very high amplitude and low amplitude sEJPs (as discussed above).

In mice, the sEJP amplitude and *F*_90–50_ were not significantly changed (Fig. 2), which was not expected with an uncoupling effect. Although the sEJP frequency was not significantly greater across all preparations, a significant increase in sEJP frequency was found in 5 out of 6 tissues. Give the occurrence of occasional spontaneous [Ca^2+^] transients in nerve terminals of the mouse (similar to those following field stimulation) it is possible that events considered as sEJPs are produced when spontaneous nerve terminal action potentials evoke synchronous (multipacketed) transmitter release. If the same phenomenon occurs in guinea pig terminals, this could explain the increase in sEJP amplitude and sEJP frequency.

An additional action of 18βGA (10 μM) seems to be on ionic currents to affect the resting *E*_m_. In both mouse and guinea pig vasa deferentia a significant depolarisation was observed (Fig. 1). The effect on resting *E*_m_ is consistent with findings in vascular (Matchkov et al., 2004) and gastrointestinal (Takeda et al., 2005) smooth muscle. This postjunctional action affecting basal electrical activity of cells might account for part of the decrease in EJP amplitude caused by 18βGA.

We suggest that 18βGA (10 μM) produces its actions through a combination of gap junction uncoupling and prejunctional effects. Whether this is an effect on prejunctional hemichannels (containing connexin or pannexin molecules) on the nerve terminal, or whether there is a different prejunctional target, has not been determined. This particular question might be best addressed through the use of connexin-specific antibodies (Mather et al., 2005).

### 4.3 The utility of gap junction blockers

Despite the increase in *R*_in_ induced by 18βGA it is surprisingly still the case that the time course of sEJP is unaltered. The most parsimonious explanation for this is that currently available gap junction blockers can influence electrical coupling between smooth muscle bundles (syncitial groups; and hence change *R*_in_), but that the more intimate local communication within bundles remains resistant to currently available drugs. This conclusion has also been made previously regarding the action of heptanol (Manchanda and Venkateswarlu, 1999). The uncoupling of syncitial groups might be functionally useful for abolishing macroscopic co-ordinated contraction, but ineffective at removing local contraction, of which micromotion in the urinary bladder is a functionally important example (Drake et al., 2005). A change in the sEJP time course, in organs like the guinea pig vas deferens, should be considered as a gold standard for detecting drugs that affect intimate coupling within smooth muscle bundles, a challenging but worthwhile pharmacological target.

## Conflicts of interest

None.

## Appendix A

The capacitance (*C*) can be defined in terms of the charge separated (*Q*) over the voltage (*V*) as $C=\frac{Q}{V}$, but as *C* is constant $C=I/\frac{dV}{dt}$ during the initial phase of current injection. During the current injection of 0.5 nA, the rate of change of voltage was 1.6 V s^− 1^, so the effective capacitance (*C*) was 0.3125 nF. The time constant of a membrane (*t*) in the product of the resistance and capacitance (*R*_m_*C*), so $R_{m}=\frac{\tau }{C}$ so R_m_ is 864 MΩ.

The total input resistance (*R*_in_) depends on both membrane resistance and junctional resistance, adding in parallel, so:

$$\frac{1}{R_{in}}=\frac{1}{R_{j}}+\frac{1}{R_{m}}\;or\;R_{j}=\frac{R_{m}R_{in}}{R_{m}-R_{in}}$$

so in this case the junctional resistance is calculated to be 9.71 MΩ. Notice that the junctional resistance is much lower than the membrane resistance, as required by Purves (1976) to allow current injection to monitor gap junction coupling.

In the mouse vas deferens we can similarly calculate the R_j_ = 26 MΩ, using *R*_in_ = 24.1 MΩ and *R*_m_ = 331 MΩ; again the junctional resistance is much lower than the membrane resistance.

## Appendix B Supplementary data

A field of OGB-1 loaded nerve terminals in the mouse vas deferens in the presence of 18βGA without EFS. Asynchronous Ca^2+^ transients are seen in many, but not all, nerve terminals. Some of the terminal branches respond synchronously, a property that allows distant, apparently unconnected branches to be tentatively identified as part of the same neuron. The movie plays at twice real time.
