# Supplementary figures and images for: Prejunctional and postjunctional actions of heptanol and 18β-glycyrretinic acid in the rodent vas deferens
Source: Auton Neurosci. 2009 Jun 15;148(1-2):69–75. doi: 10.1016/j.autneu.2009.03.006 (PMC2758546; doi:10.1016/j.autneu.2009.03.006)

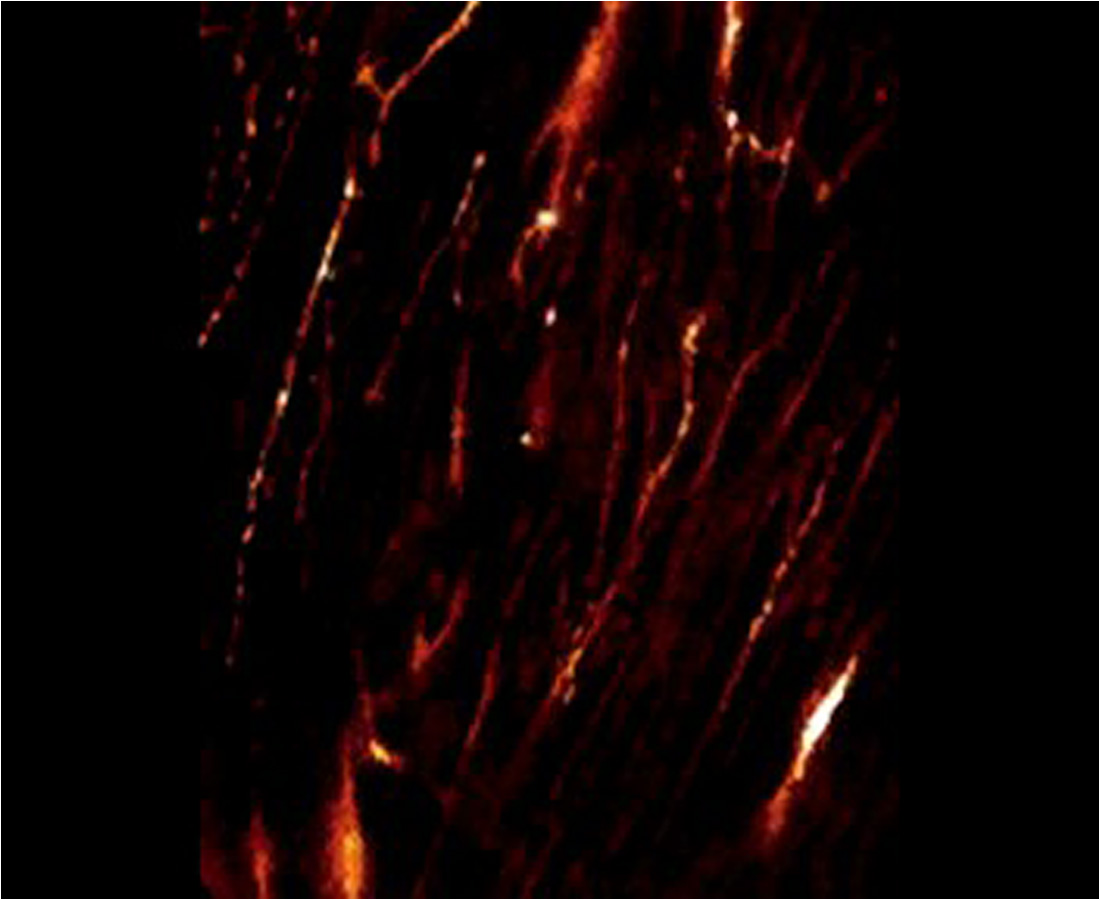

Supplement: Supplementary file 1 — A field of OGB-1 loaded nerve terminals in the mouse vas deferens in the presence of 18βGA without EFS. Asynchronous Ca2+ transients are seen in many, but not all, nerve terminals. Some of the terminal branches respond synchronously, a property that allows distant, apparently unconnected branches to be tentatively identified as part of the same neuron. The movie plays at twice real time. [file mmc1.jpg]
